# Scar site metastasis of renal cell carcinoma diagnosed on-site cytology: a case report

**DOI:** 10.1186/s12885-018-4167-2

**Published:** 2018-03-07

**Authors:** Santosh Tummidi, Pragati Sathe, Prachi Gholap, Manoj Patil, Kanchan Kothari

**Affiliations:** Department of Pathology, Seth GSMC & KEMH, Parel, Mumbai, 400012 Maharashtra India

**Keywords:** Renal cell carcinoma, Fine needle cytology, Scar recurrence, Toluidine blue, Nephrectomy

## Abstract

**Background:**

Renal cell carcinomas (RCCs) have a propensity for widespread metastases and a wide range of survival rates. They can spread into adjacent organs by direct extension and can invade local or distant sites by lymphatic, hematogenous or lympho-hematogeneous pathways. Scar site metastasis is very rare.

**Case presentation:**

We report a rare case of scar site RCC metastasis in a patient who underwent left radical nephrectomy 10 months ago.

**Conclusion:**

FNAC is a simple and easy technique that can help in the definitive diagnosis of subcutaneous lesions. A correct early stage diagnosis of metastatic RCC can considerably improve the survival rates.

## Background

Renal cell carcinoma (RCC) is one of the most aggressive genitourinary cancers with unpredictable and diverse behavior [1]. There has been an increase in the incidence of RCCs over the last 2 decades [2]. About 30–50% of patients were found to have metastases by the time of diagnosis. Bone, lymph nodes, lungs, and brain constitute expected ‘homing’ sites, whereas metastasis may turn up at unusual locations too [3].

Scar site metastasis after nephrectomy is very rare with only a few cases reported in the literature [1]. Fine needle aspiration (FNA) is an excellent tool for early diagnosis of subcutaneous nodules which in presence of characteristic cytomorphology excludes the need for any invasive methods [4]. We report a case of scar site metastasis diagnosed on FNA.

## Case presentation

A 68-year-old male, presented with complaints of swelling over the left lower abdominal wall for 1 month. The swelling was along the scar of radical nephrectomy that had been done 10 months ago for renal cell carcinoma. The primary tumor on histology was a conventional (clear cell) RCC with Fuhrman nuclear grade 2 and there was involvement of renal capsule and gerota’s fascia with extension up to the perinephric fat.

**Fig. 1:**
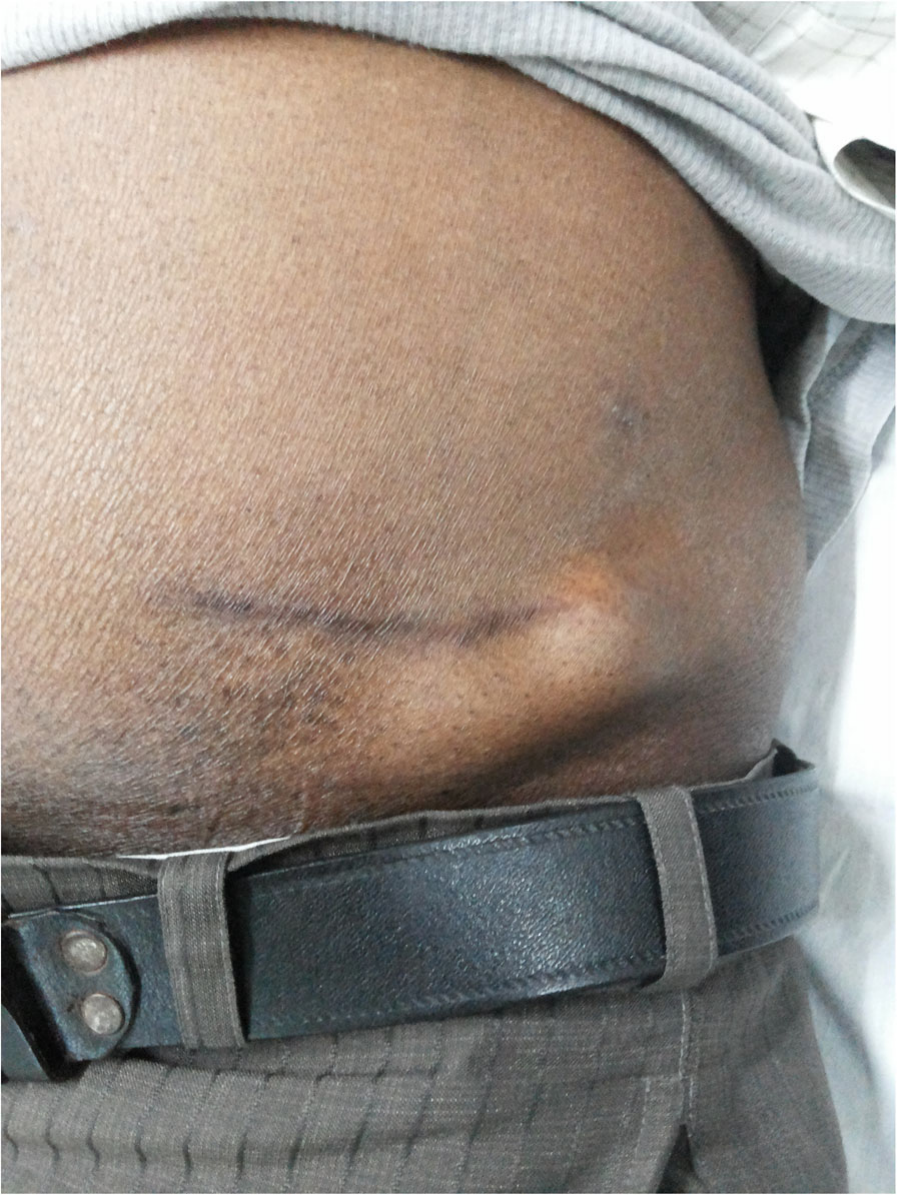
Sub cutaneous swelling over the left iliac region near the previous scar site

On examination, the patient had a well-defined, soft, non-tender, reddish non-pulsatile cystic swelling over the left lumbar region near the scar mark. Figure 1 He had no inguinal lymphadenopathy. USG abdomen pelvis revealed two well-defined hypo-echoic to iso-echoic lesions in the lateral abdominal wall measuring 19 × 16 mm and 18 × 16 mm at scar site with no evidence of vascularity within the scar. Radiological impression was a keloid. FNA from the swelling was done and screened onsite for adequacy by toluidine blue stain. Routine Papanicolaou (PAP) and Giemsa stains were also performed.

**Fig. 2:**
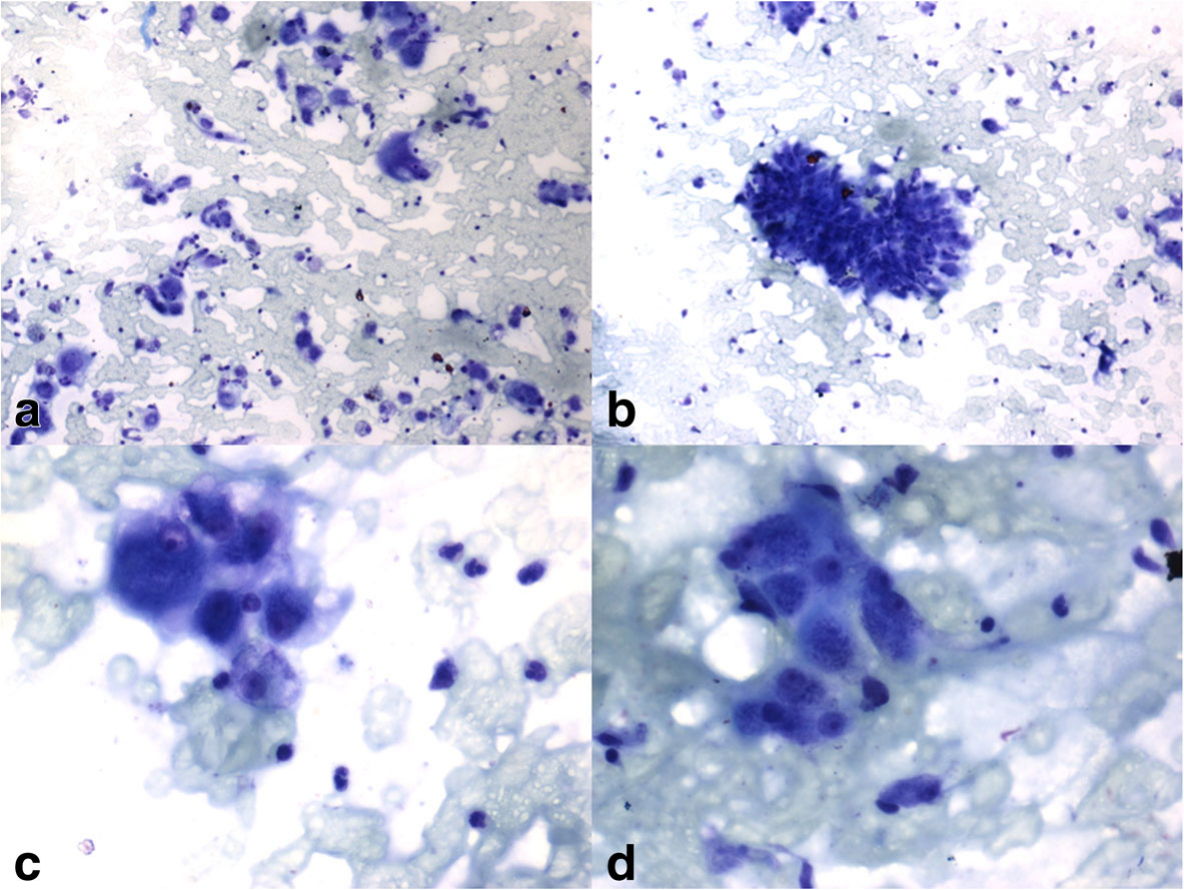
**a** Smears were blood mixed with malignant epithelial cells scattered singly and in clusters (**b**). **c, d** Tumor cells with round to polygonal shape, eccentrically placed hyperchromatic nuclei & prominent nucleoli. Cytoplasm was abundant and granular eosinophilic (TB, × 10, × 40)

**Fig. 3:**
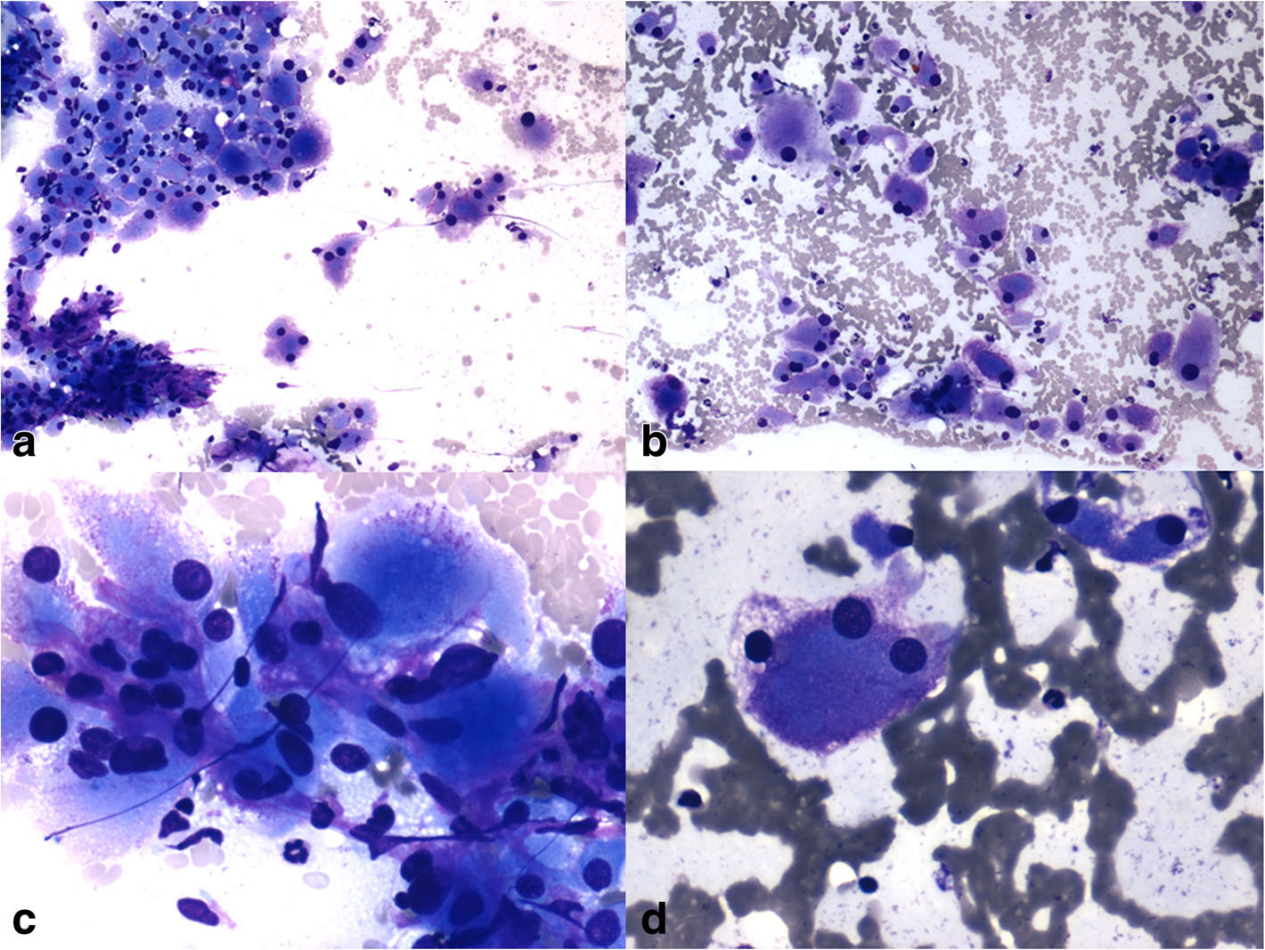
**a** Cytosmears were blood mixed with malignant epithelial cells scattered singly and in clusters (**b**). **c**, **d**: Round to polygonal shaped tumor cells with eccentrically placed hyperchromatic nuclei & prominent nucleoli. Cytoplasm was abundant and granular eosinophilic (Giemsa, × 10, × 40)

**Fig. 4:**
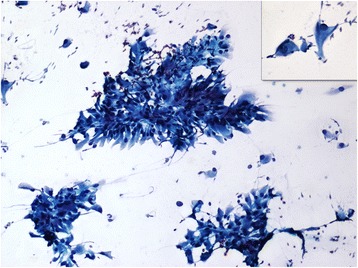
Cytosmears malignant epithelial cells predominantely in clusters around a fibrovascular core. Round to polygonal tumor cells with eccentrically placed hyperchromatic nuclei & prominent nucleoli. Cytoplasm was abundant and granular (Inset) (PAP, × 10, × 40)

Smears were hemorrhagic and showed epithelial cells in loose clusters and scattered singly. The tumor cells had a round to polygonal shape with eccentrically placed hyperchromatic nuclei and prominent nucleoli. Cytoplasm was abundant and granular, eosinophilic to vacuolated. The clusters showed delicate, fibrillary fibrovascular cores. A few bizarre cells were also seen. Figures. 2,3,4 With the above morphology that was characteristic of RCC and the history, a diagnosis of metastatic renal cell carcinoma to the scar site was given. Immunocytochemistry showed CD10 positivity in tumor cells. At last, follow up the patient had expired 6 months after FNA, he had taken no further treatment due to his poor socioeconomic condition.

## Discussion and conclusions

RCCs are a heterogeneous group of tumors with 1/3rd of patients already having distant metastases at the time of diagnosis and in 1/4th of patient’s metastasis occurs after radical nephrectomy. The tumors frequently metastasize to the lung (50–75%), bone (30–40%), liver (30–40%), brain and thyroid (25%) [1]. This event usually occurs after many years but could be the presenting feature of underlying cancer. Scar site metastasis following conventional radical nephrectomy is very rare. In recent years the numbers of laparoscopic nephrectomies have increased, raising the doubt regarding the oncologic safety of this approach, especially regarding local or port-site metastasis. However, randomized studies have not been able to show any significant difference in the incidence of scar-site metastases (0.9–1.8%) following conventional open nephrectomies and port-site metastases following laparoscopic nephrectomies [5]. Pathogenesis of scar-site metastases from RCC is multifactorial. The reported gap for recurrence is 6 months to 5 years after initial diagnosis/nephrectomy [4, 6]. Major contributing factors include natural tumor behavior, local wound factors, and immunity. Following open nephrectomies, the most important factor for tumor cell dissemination has been improper techniques/manipulation [7]. Our patient underwent a conventional radical nephrectomy and there was no documentation of any tumor spillage during surgery.

The cytological differentials of scar site metastasis of RCC include histiocytic cells arranged singly or in groups as the cells have abundant vacuolated cytoplasm with a low nuclear cytoplasmic ratio. Nucleoli may be prominent depending on the Fuhrman grade of the tumor. Careful attention to the nuclear morphology and the presence of delicate fibrillary vascular cores between tumor cells aids correct diagnosis. Other differentials include fibrohistiocytic lesions of the skin and subcutaneous tissue (xanthomatous dermatofibroma, MFH, plexiform fibrohistiocytic tumor, xanthoma, and granular cell tumor) and various skin adnexal tumors (sebaceous hyperplasia, sebaceous adenoma and carcinoma, clear cell sarcoma, clear cell syringoma, clear cell hidradenoma and clear cell porocarcinoma) [8].

Immunochemistry can play an essential role in differentiation of the malignant cells in metastatic RCC at subcutaneous tissue from the other benign/malignant lesions of skin. Markers such as RCCma, CD10, EMA and PAX 8 suggest renal origin [6]. The other markers which are helpful include histiocytic markers (CD68 and lysozyme) in fibrohistiocytic lesions; CD34 and Factor XIIIA in dermatofibroma; keratin and EMA for sebaceous carcinoma; S100, HMB-45, and Melan-A for clear cell sarcoma [4, 6].

Management for local recurrence includes conservative treatment, surgery, radiotherapy or combination treatments [9]. Overall, the occurrence of subcutaneous metastases portends a poor outcome.

Subcutaneous scar site metastases from RCC are uncommon and typically imply a very poor prognosis and short survival. FNA can be a rapid and quick technique for detection of the metastatic lesion at superficial subcutaneous sites. Complete clinical history with immunocytochemistry if needed is useful for diagnosis.

## Abbreviations

CD: Cluster of differentiation
EMA: Epithelial membrane antigen
FNA: Fine needle aspiration
HMB: Human melan black
PAP: Papanicolaou
PAX: Paired box gene
RCC: Renal cell carcinoma
SMA: Smooth muscle actin
